# Clinical determinants of psychiatric care in genetic neurodevelopmental disorders: a cross-sectional analysis

**DOI:** 10.1186/s11689-025-09654-0

**Published:** 2025-10-07

**Authors:** David J. Adams, Alexandra M. Klomhaus, Nicole R. Wong, Benjamin N. Schneider, Charlotte DiStefano, Sunil Mehta, Rujuta B. Wilson, Julian A. Martinez-Agosto, Shafali S. Jeste, Aaron D. Besterman

**Affiliations:** 1 Massachusetts General Hospital, Boston, MA USA; 2 McLean Hospital, Belmont, MA USA; 3 Harvard Medical School, Boston, MA USA; 4 David Geffen School of Medicine, Department of Medicine Statistics Core, University of California Los Angeles, Los Angeles, CA USA; 5 Department of Psychiatry and Behavioral Sciences, The Johns Hopkins University School of Medicine, Baltimore, MD USA; 6 Semel Institute for Neuroscience and Human Behavior, University of California Los Angeles, Los Angeles, CA USA; 7 Department of Psychiatry, Division of Child and Adolescent Psychiatry, University of California Los Angeles, Los Angeles, CA USA; 8 Department of Psychiatry, Children’s Hospital Los Angeles, Los Angeles, CA USA; 9 Department of Psychiatry & The Biobehavioral Sciences, University of Southern California, Los Angeles, CA USA; 10 PrairieCare Medical Group, Rochester, MN USA; 11 David Geffen School of Medicine, University of California Los Angeles, Los Angeles, CA USA; 12 Department of Human Genetics, University of California Los Angeles, Los Angeles, CA USA; 13 Division of Neurology, Children’s Hospital Los Angeles, Los Angeles, CA USA; 14 Departments of Pediatrics and Neurology, University of Southern California, Los Angeles, CA USA; 15 Department of Psychiatry, Division of Child and Adolescent Psychiatry, University of California San Diego, San Diego, CA USA; 16 Laura Rodriguez Research Institute of the Family Health Center of San Diego, San Diego, CA USA

**Keywords:** Neurodevelopmental disorders, Neuropsychiatry, Precision medicine, Genetic testing, Multidisciplinary care

## Abstract

**Background:**

This study aims to identify clinical and developmental factors associated with psychotropic medication exposure and subspecialty psychiatric service utilization among patients with genetic neurodevelopmental disorders (GNDDs).

**Methods:**

We conducted a retrospective analysis of 316 patients from the Care and Research in Neurogenetics (CARING) Clinic at the University of California, Los Angeles (UCLA). We assessed the association between neurodevelopmental and psychiatric diagnoses, behavioral histories, family history, and service utilization with two outcomes: (1) the number of psychotropic medication classes trialed before clinic intake and (2) whether the patient was evaluated by a CARING psychiatrist. Poisson and logistic regression models were used to evaluate associations while adjusting for demographic and clinical covariates.

**Results:**

Individuals with more severe behavioral disturbances had higher psychiatric service needs, while intellectual disability was associated with greater psychotropic medication exposure but not increased psychiatric consultation, possibly due to prior community-based care. The presence of a pathogenic/likely pathogenic genetic variant was not associated with either outcome, suggesting that genetic diagnosis alone does not predict psychiatric needs. Instead, behavioral comorbidities, not genetic status, were the primary drivers of psychotropic use and psychiatric referrals. A history of developmental delay was negatively associated with psychiatric consultation, and mediation analyses indicated that early intervention services partly explained this relationship. Additionally, patients receiving behavioral therapies had higher psychotropic exposure, reflecting greater clinical complexity and frequent use of multimodal treatment strategies.

**Conclusions:**

Our findings suggest that psychiatric needs in GNDDs are more closely tied to behavioral comorbidities than to genetic diagnosis status, reinforcing the importance of symptom-driven psychiatric evaluation. The observed relationship between early developmental interventions and psychiatric service utilization warrants further longitudinal investigation. These results highlight opportunities to optimize psychiatric care pathways through early screening, integrated behavioral and pharmacologic interventions, and targeted resource allocation for individuals with neurodevelopmental disorders.

**Supplementary Information:**

The online version contains supplementary material available at 10.1186/s11689-025-09654-0.

## Background

Behavioral comorbidities, including inattention, hyperactivity, obsessions, compulsions, anxiety, irritability, and depressed mood, are common in individuals with autism spectrum disorder (ASD), intellectual disability (ID), and global developmental delay (GDD) [1–3]. Treatment often requires a multimodal approach that combines educational interventions, behavioral therapies, and psychotropic management [4–6]. Although practices vary, in recent decades, psychotropic medication prescribing for patients with neurodevelopmental disorders (NDDs) has increased globally [7–19]. Many individuals with NDDs that are prescribed psychotropic medications have high levels of morbidity and functional impairment that are non-responsive to behavioral intervention alone, justifying the risk of psychotropic medications [5, 14]. However, other individuals with NDDs are on complex, longstanding, multidrug psychotropic regimens with unclear indications and, sometimes, without ever being evaluated by a psychiatrist. Given the potential for serious long-term side-effects and dangerous drug-drug interactions from such regimens, it is important to understand the specific factors associated with increased psychotropic exposure in patients with NDDs [5, 14, 15]. 

There are many factors that may play a role in determining psychiatric comorbidities and subsequent psychotropic exposure. For example, psychotropic prescribing patterns vary by sex, socioeconomic status (SES), and the specific class of psychotropic medication prescribed [11, 19–23]. Females are predominantly prescribed antidepressants and mood stabilizers, while males are predominantly prescribed stimulants and antipsychotics [20]. Alternatively, the specific genetic architecture underlying each NDD may influence the type or severity of comorbidities and psychotropic treatment patterns, although considerable phenotypic heterogeneity exists even within genetically defined conditions. The impact of having a rare, pathogenic or likely pathogenic (P/LP) genetic variant on psychotropic exposure for patients with NDDs has not been widely explored. P/LP variants can be detected in up to half of those severely affected by NDDs [24–26]. We previously showed in this cohort that patients with a P/LP variant were more likely to be female and have a history of motor delay, hypotonia, congenital heart disease, and early intervention [27]. We now compare the overall burden of specific psychiatric and behavioral problems between P/LP and ‘idiopathic’ cohorts (i.e., individuals with NDDs without an identifiable P/LP variant) and hypothesize that individuals with genetic NDDs (GNDDs) may have greater psychiatric and behavioral problems than those with idiopathic NDDs, potentially leading to higher psychotropic exposure. However, we recognize that psychiatric vulnerability can also be high in idiopathic cases due to a range of common genetic variation, as well as epigenetic and environmental factors, such as in the cases of prenatal exposures or maternal infections.

We leverage our unique cohort of patients from the University of California, Los Angeles (UCLA) Care and Research in Neurogenetics (CARING) Clinic to describe the clinical profile associated with psychotropic exposure in the GNDDs population. We further describe the variables associated with being evaluated by a psychiatrist in the CARING Clinic to more clearly elucidate the profile of patients requiring this level of subspecialty psychiatric care.

## Methods

The present study was conducted with the approval of the UCLA Medical Institutional Review Board (IRB) 3. The study was conducted in adherence to the Strengthening the Reporting of Observational Studies in Epidemiology (STROBE) guidelines for observational studies. Complete details on patient recruitment and characteristics are described elsewhere [27, 28]. In brief, subjects were patients seen for intake in the UCLA CARING Clinic between January 1, 2014, and January 1, 2019. The CARING clinic provides subspecialty services in medical genetics, neurology, psychiatry, and psychology for patients with known or suspected GNDDs [27–29]. Patients were referred for evaluation by clinicians, patient advocacy groups, and research studies. CARING providers often brought their own patients from other settings into the clinic for further multidisciplinary assessment and treatment. As the clinic became more well known in the community, independent research and word of mouth resulted in an increasing number of families self-referring. Upon intake, patients were cared for by one or more specialists in neurology, medical genetics, and psychiatry. Each family’s unique needs dictated which specialists were involved in care. Some patients were seen for one evaluation; others received ongoing assessment and care over several monthly visits. In addition to seeing the physicians in our clinic, some patients also received care from providers in clinical psychology and social work in our clinic.

To be evaluated in the clinic, a patient had to have a known or suspected GNDD. Genetic diagnoses were established through clinical testing ordered prior to or during intake at the multidisciplinary NDD clinic, as described previously [27, 28]. Testing modalities varied by patient and included chromosomal microarray (CMA), single-gene testing, multigene panels, and exome sequencing. CMA was typically performed using SNP-based arrays (e.g., Illumina Infinium or Affymetrix CytoScan HD), but platform details varied based on ordering institution and time period. Exome sequencing and gene panels were conducted at CLIA-certified laboratories and interpreted using ACMG-AMP guidelines. Because this was a retrospective real-world cohort, genetic testing was not performed uniformly across all patients; rather, it reflected the evolving clinical standard of care between 2014 and 2019. Diagnostic variants were manually confirmed and classified as pathogenic or likely pathogenic by licensed genetic counselors or medical geneticists at the time of review. Full diagnostic yield, gene lists, and platform descriptions are available in prior publications [27, 28]. Most GNDD patients were initially identified based on clinical suspicion (i.e., based on syndromic presentation) with subsequent confirmation of a P/LP variant through molecular testing obtained either before or after clinic intake. Patients without a P/LP variant identified on diagnostic genetic testing were considered to have an idiopathic NDD for further analysis. The most common genetic diagnoses in the cohort were duplication 15q syndrome, tuberous sclerosis complex, Angelman syndrome, and 15q11.2 BP1–BP2 microdeletion syndrome. A full list of recurrent diagnoses is available elsewhere [28]. 

Upon establishing care with the CARING clinic, most patients already carried a diagnosis of ASD, ID, or both. Formal neuropsychological testing was rarely conducted because we did not have regular access to neuropsychologists. However, a meticulous clinical and developmental history was obtained for each patient. From those histories and expert clinical assessment, we were able to confirm, establish, or adjust clinical diagnoses of ASD and ID by retrospectively applying DSM-5 diagnostic criteria and, when available, reviewing neuropsychological testing results previously obtained in other settings. We also performed a thorough assessment of motor and language milestones to diagnose developmental delays. For the remainder of this paper, we define developmental delay (DD) specifically as a history of delayed motor and/or language milestones as documented in clinical records. This definition was chosen intentionally to reflect the types of delays most consistently reported and recognized by clinicians in our real-world cohort. We do not use the term to imply a diagnosis of Global Developmental Delay, which would require standardized evaluation across multiple developmental domains. Service history was reviewed for documentation of behavioral, speech, occupational, or physical therapy. Early intervention was defined as receipt of one or more of these services initiated before age of three, based on documentation in the medical record. Psychiatric diagnoses were extracted from the electronic medical record and reflected clinical impressions documented by treating psychiatrists or neurologists. Standardized diagnostic instruments (e.g., structured interviews or validated questionnaires) were not uniformly used, and diagnoses were based on expert clinical judgment.

The legal guardians of one-hundred-ten patients provided informed consent for prospective collection of clinical data (UCLA IRB protocol: 14-001908). When possible, patient assent was also obtained. With an IRB-approved waiver of consent, the charts of 206 additional patients were retrospectively reviewed to increase our cohort size (UCLA IRB protocol: 19–000121). Patient data were manually extracted from the UCLA electronic medical record into a deidentified, encrypted, HIPAA-compliant database. Subsequent analyses were performed using the coded, deidentified data.

We explored potential differences in phenotypic and clinical profiles between those with GNDDs and idiopathic NDDs. We also explored how elements from patients’ psychiatric histories, neurodevelopmental histories, genetic testing results, service use histories, medical comorbidities, and family histories were associated with (1) the number of psychotropic class exposures prior to intake and (2) whether a patient was ever evaluated by a CARING Clinic psychiatrist (Table S1). Because age dictates the time frame for possible psychotropic exposure and the likelihood of benefitting from a psychiatric evaluation, we controlled for patient age at intake (in months). Because other demographic factors are known to be associated with access to care – and, subsequently, ability to receive psychotropic prescriptions – we controlled for potential confounders, including sex assigned at birth, race (white versus not), SES (using Area Deprivation Index national percentiles generated with the Neighborhood Atlas ^®^), and primary insurance status (commercial versus public {i.e., Medicaid or Medicare} versus no documented coverage) [30]. For models that considered the number of psychotropic class exposures as the outcome, we also controlled for lifetime history of comorbid seizure disorder given the expected use of benzodiazepines or antiepileptic drugs in this group separate from any psychiatric indication.

### Statistical analysis

We present demographic data, including means and standard deviations for continuous variables and counts and frequencies for categorical variables, both overall and stratified by NDD type (Table 1). We also present descriptive statistics for all variables, both overall and stratified by whether the patient was seen by CARING psychiatry (Table S1). To detect possible differences in phenotypic and clinical profiles, we compared the frequencies of categorical variables between those with GNDDs and idiopathic NDDs using chi-square analyses (Table S2).

**Table 1:** Descriptive statistics of covariates by type of NDD

	All Patients (*N* = 316)		Genetic NDD (*N* = 152)		Idiopathic NDD (*N* = 164)	
Continuous Covariates	*M (Range)*	*SD*	*M (Range)*	*SD*	*M (Range)*	*SD*
Age at Intake (Months)	118.99 (0-535)	92.69	111.61 (0-407)	96.61	125.84 (2-535)	88.65
Area Deprivation Index (National Percentile)	9.63 (1–94)	11.9	8.12 (1–53)	8.8	11.02 (1–94)	14.04

	All Patients (***N*** = 316)		Genetic NDD (***N*** = 152)		Idiopathic NDD (***N*** = 164)	
Categorical Covariates	*N*	*%*	*N*	*%*	*N*	*%*
Sex (Male)	208	65.82	81	53.29	127	77.44
Sex (Female)	108	34.18	71	46.71	37	22.56
Ethnicity (White)	178	56.33	85	55.92	93	56.71
Primary Insurance Status (Commercial)	217	68.67	102	67.11	115	70.12
Primary Insurance Status (Public)	73	23.1	39	25.66	34	20.73
Primary Insurance Status (No Insurance Documented)	26	8.23	11	7.24	15	9.15

For our outcome of number of psychotropic class exposures prior to intake, a count variable, we used Poisson regression to individually assess each factor of interest while controlling for abovementioned confounding covariates. We present incident rate ratios (IRRs), corresponding 95% confidence intervals (CIs), and p-values (both raw and adjusted for multiple comparisons; Table S3).

For our outcome of evaluation by a CARING Clinic psychiatrist, a binary variable, we used logistic regression to similarly assess each individual factor while controlling for abovementioned confounding covariates. We present odds ratios (ORs), 95% CIs, and p-values (both raw and adjusted for multiple comparisons; Table S4).

We included a post-hoc interaction term between applied behavior analysis (ABA) and a subset of factors, namely internalizing and externalizing disorders, schizophrenia spectrum and other psychotic disorders, ASD, ID, and DD, to test for a moderating effect of ABA therapy. In the absence of significant interaction effects, we present results from models excluding the interaction term.

Lastly, post-hoc, we examined early intervention (EI) as a mediator between DDs and seeing a CARING Clinic psychiatrist by running a series of regression models: (1) seeing a CARING psychiatrist on DDs, (2) EI on DDs, and (3) seeing a CARING psychiatrist on DDs and EI. We then assessed change of effect of DD on seeing a CARING psychiatrist in models with and without EI.

To adjust our p-values for multiple comparisons, we used the Benjamini-Hochberg false discovery rate (FDR) correction. All analyses were conducted using SAS 9.4 (SAS Institute, Cary, NC) and RStudio (2023).

## Results

**Fig. 1:**
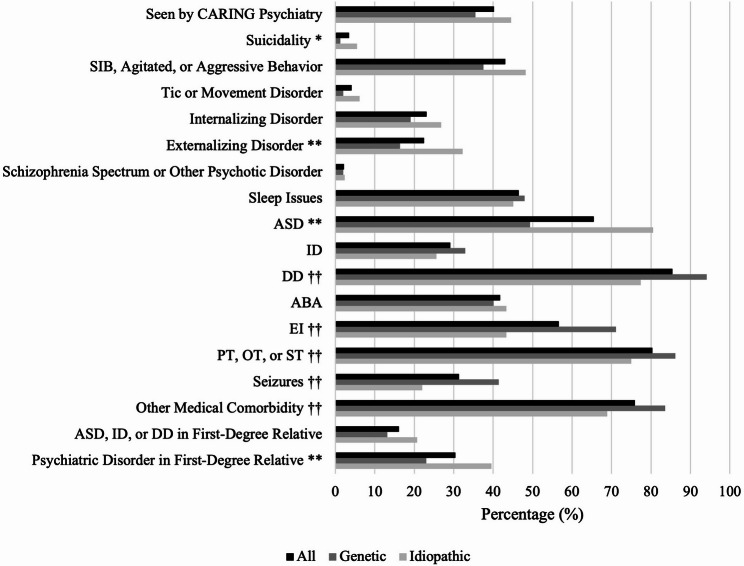
Phenotypic Comparison Between GNDDs and Idiopathic NDDs. Percentage of patients with GNDDs and idiopathic NDDs with positive history of each categorical variable. Categorical variables with statistically higher frequency in the idiopathic group are marked with asterisks (*). Those with one asterisk have an uncorrected p-value < 0.05, those with two asterisks have a corrected p-value < 0.05 (i.e., survived FDR correction). Categorical variables with statistically higher frequency in the GNDD group are marked with obelisks. All are marked with two obelisks as all had a corrected p-value < 0.05 (i.e., survived FDR correction). See Supplemental Tables 2 and 3 for more details on chi-square results and for descriptions of continuous variables by group

We compared the phenotypic and clinical profiles between those with GNDDs and those with idiopathic NDDs using a chi-squared test. We found that those with GNDDs had a lower frequency of suicidality and ASD than those with idiopathic NDDs (Fig. 1, Table 1, Table S2). Interestingly, those with GNDDs also had lower frequency of psychiatric illness within a first-degree relative. One possible explanation for the lower rate of psychiatric illness in first-degree relatives of GNDD patients is that many of these diagnoses may arise from de novo mutations, which are less likely to be inherited. However, genetic testing in this cohort was not uniform, and exome sequencing was not universally performed, limiting our ability to detect inherited variants with incomplete penetrance or variable expressivity. Although our prior work [27] did not directly assess family psychiatric history in ES-negative cases, the possibility of undetected transmitted variants remains an important consideration. Those with GNDDs also had higher frequency of DD, seizures, and medical comorbidities. They were also more likely to have used EI, physical therapy (PT), occupational therapy (OT), or speech therapy (ST) prior to intake. All but one significant finding (suicidality) survived FDR correction. The phenotypic and behavioral profiles were otherwise similar between the GNDD and idiopathic groups. Notably, having a GNDD was not associated with psychotropic exposure nor being seen by a CARING psychiatrist.

**Fig. 2:**
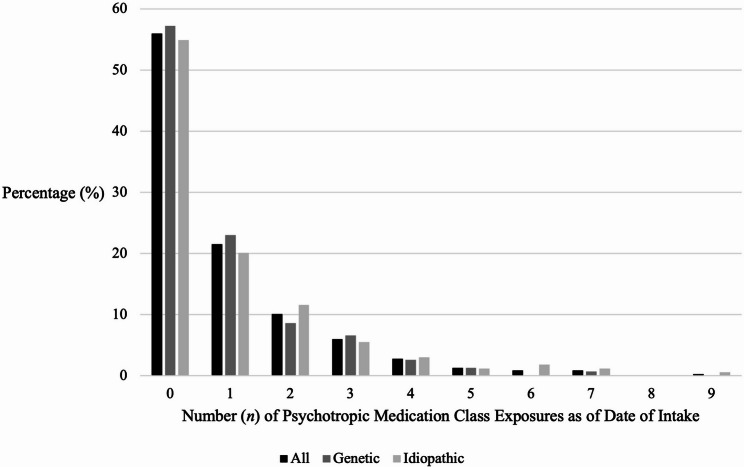
Psychotropic medication class exposures between GNDDs and Idiopathic NDDs. Total number of psychotropic medication class exposures as of the date of intake. Bars represent percentage of each group (all {*n* = 316}, GNDD {*n* = 152}, and idiopathic {*n* = 164}) who trialed each number of classes

The mean number of psychotropic class exposures by clinic intake was 1.85 (*SD* = 2.08; Fig. 2, Tables S5 and S6), and 127 patients (40.2%) were eventually seen by a CARING psychiatrist. We compared the total number of psychotropic medication classes between groups using a Wilcoxon two-sample test. Psychotropic medication class exposure prior to intake was higher among patients evaluated by CARING Clinic psychiatrists compared to patients not evaluated by CARING Clinic psychiatrists, with average exposures of 2.6 and 1.4, respectively (*p* < 0.0001).

**Fig. 3:**
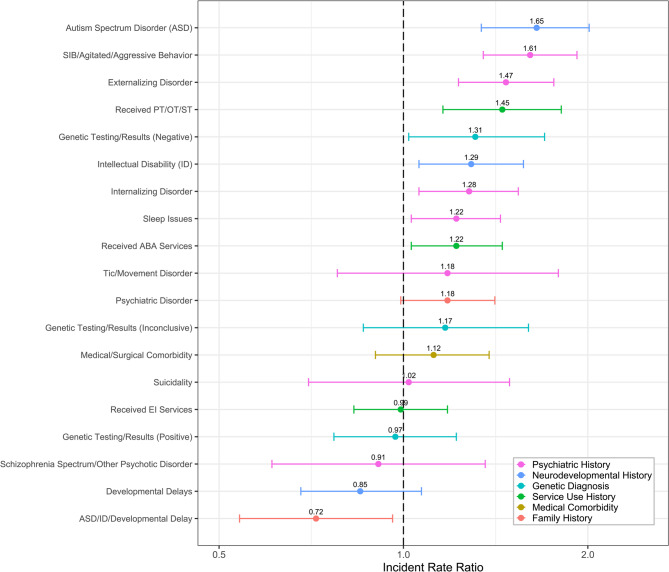
IRRs of poisson regression models with psychotropic exposure outcome

We found numerous significant associations between clinical factors and psychotropic class exposures prior to intake (Fig. 3, Table S3). Notably, we found that those with history of self-injurious behavior (SIB) or agitation, sleep disorders, internalizing disorders, externalizing disorders, ASD, and ID had higher exposure rates (Fig. 3). Additionally, those with history of using ABA or PT/OT/ST services had higher psychotropic exposure rates. All but one significant finding (genetic results negative vs. undocumented) survived FDR correction, suggesting robust associations between selected factors and psychotropic class exposure.

**Fig. 4:**
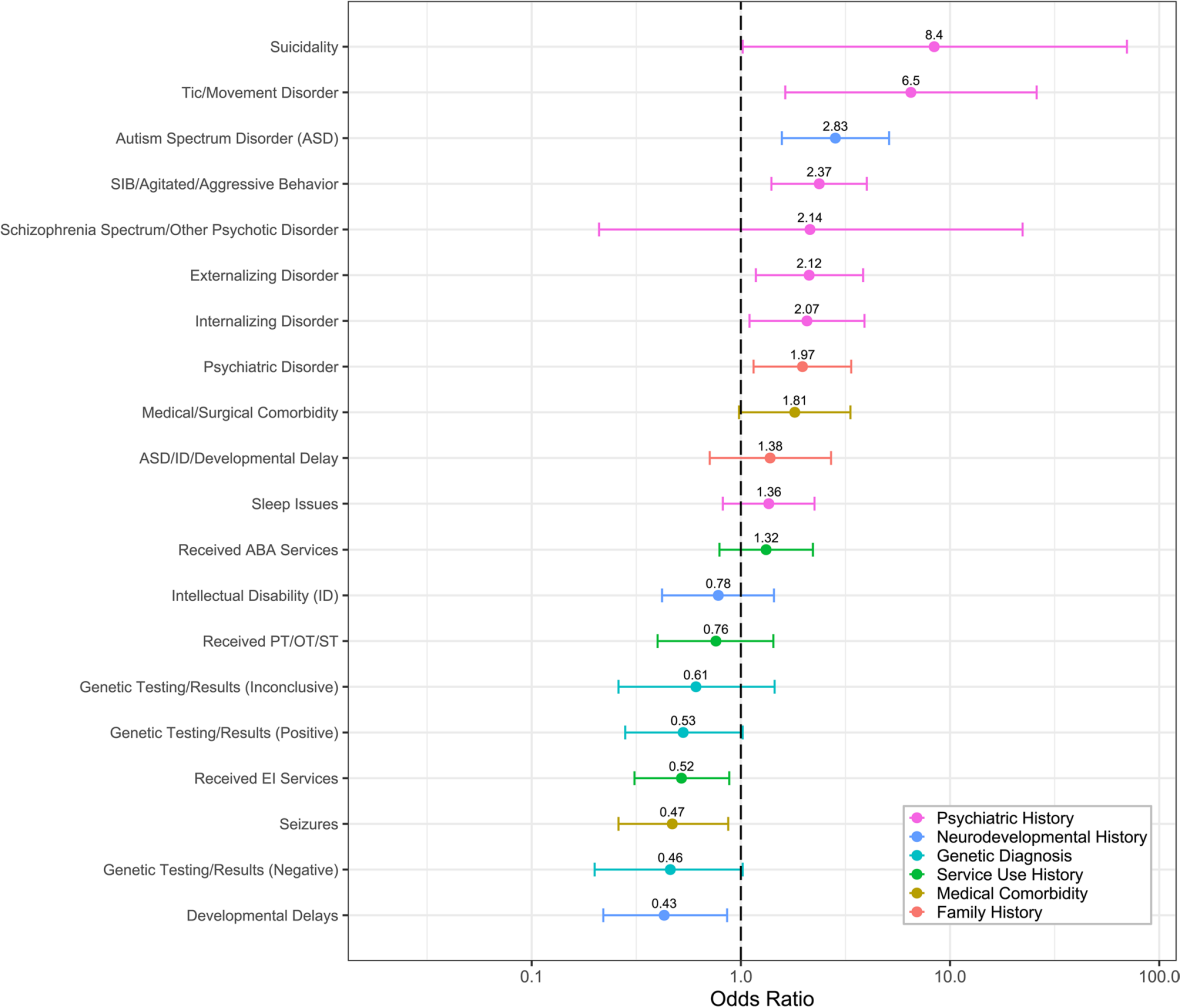
Title. ORs of bivariate logistical regression models with CARING psychiatry evaluation outcome

We found similar associations between clinical factors and being seen by a CARING psychiatrist (Fig. 4, Table S4). Those with history of suicidality, SIB or agitation, movement disorders, internalizing disorders, externalizing disorders, and ASD were more likely to be seen by a CARING psychiatrist. Those with history of DD and those with history of using EI services were less likely to have received an evaluation from a CARING psychiatrist. While only two associations (i.e., history of SIB, agitated, or aggressive behavior and history of ASD) survived FDR correction, many associations only narrowly failed to maintain statistical significance.

### Moderator and mediator analyses for CARING psychiatry outcome

We did not find a significant interaction between history of receiving ABA therapy and internalizing disorders (*p* = 0.3188), externalizing disorders (*p* = 0.5145), ASD (*p* = 0.7599), ID (*p* = 0.4220), or DD (*p* = 0.1245).

Assessment of EI as a mediator suggested partial mediation of the negative relationship between DD and seeing a CARING psychiatrist. In univariate logistic regression models that did not control for other covariates, we found that DD was negatively associated with seeing a CARING psychiatrist (OR = 0.37, *p* = 0.0025), DD was positively associated with having received EI (OR = 14.9, *p* < 0.0001), and having received EI was negatively associated with seeing a CARING psychiatrist (OR = 0.38, *p* < 0.0001). Including EI in a regression model of seeing CARING psychiatry on DD made the relationship between DD and seeing a CARING psychiatrist disappear (OR = 0.57, *p* = 0.1063) with EI maintaining its significant association with seeing CARING psychiatry (p-0.0012). This represented a percentage mediated of 29.3% (*p* = 0.1208).

Evidence of mediation is less clear in models that account for confounders. In univariate logistic regression models that did control for other confounders, we found that DD was negatively associated with seeing a CARING psychiatrist (OR = 0.43, *p* = 0.0171), DD was positively associated with having received EI (OR = 13.9, *p* < 0.0001), and having received EI was negatively associated with seeing a CARING psychiatrist (OR = 0.52, *p* = 0.0138). Including EI in a regression model of seeing CARING psychiatry on DD made the relationship between DD and seeing a CARING psychiatrist disappear (OR = 0.54, *p* = 0.1039). However, EI lost its association with seeing CARING psychiatry in this model (*p* = 0.0828). This represented a percentage mediated of 20.5% (*p* = 0.2240).

## Discussion

In this cross-sectional study of patients with GNDDs, we identified several clinical factors that significantly influence psychotropic medication use and the engagement of subspecialty psychiatric services. As expected, our findings mirror prior research showing that individuals with NDDs who exhibit severe behavioral disturbances or poorer adaptive functioning have elevated psychiatric service needs [5, 16–19, 22, 23, 31, 32]. Interestingly, the presence of ID was associated with greater psychotropic medication exposure in our cohort, but did not correspond to an increased likelihood of subspecialty psychiatry evaluation​. This pattern may indicate that many children with ID were already receiving psychiatric care in community settings prior to their clinic intake, reducing the need for an additional specialist consultation in our clinic​. However, without prospective data, we cannot confirm this interpretation.

Notably, the presence of a P/LP genetic variant was not associated with either psychotropic medication use nor the engagement of subspecialty psychiatric services in our analysis​. While prior studies have found that a genetic cause can be identified in up to half of individuals with severe NDDs, our findings suggest that genetic testing results alone do not correlate with a patient’s psychiatric medication needs or specialty service utilization [24, 26]. This suggests that factors other than rare variants are likely underlying neurobehavioral traits, such as the collective effect of common variants (as previously shown in 22q11.2 deletion syndrome) and/or environmental factors [33, 34]. We also observed that the behavioral and clinical profiles of children with GNDDs were largely similar to those with idiopathic NDDs, with trends, if any, toward *greater* behavioral comorbidity in the idiopathic group, again suggesting that rare variants are unlikely to be the major factor driving NDD-associated behaviors.​ From a clinical perspective, this means that the mere presence of a known genetic diagnosis should not be viewed as an indicator of higher or lower psychiatric need – clinicians should base psychiatric assessment and interventions on the child’s developmental and behavioral presentation, rather than on genetic status.

One unexpected finding was that a history of DD showed a negative association with being seen by a CARING psychiatrist, even though DD was not associated with differing medication exposure. In our analysis, children with documented DD were substantially less likely to receive a psychiatric evaluation in the specialty clinic (in adjusted models, DD was associated with roughly 60% lower odds of psychiatric referral, OR ~ 0.4)​. Our exploratory mediation analyses suggest that EI services may partly explain this relationship: DD was highly correlated with prior EI service use (unadjusted OR ~ 14.9) and receiving EI was independently associated with lower odds of psychiatric consultation (adjusted OR ~ 0.5)​. When we accounted for EI use, the direct association between DD and psychiatric evaluation was attenuated and no longer significant, consistent with partial mediation​. This finding aligns with the notion that primary care providers often identify developmental delays early and refer these children to therapeutic services, which can facilitate earlier behavioral interventions and support [35–39]. Engaging in early intervention might address developmental and behavioral issues, potentially delaying or reducing the need for subspecialty psychiatric care at young ages (though the direction of causality cannot be confirmed from our data). While our data cannot speak to the specific type, intensity, or duration of these services—given that early intervention documentation was extracted from retrospective clinical notes—the consistency of the association suggests that even heterogeneous early supports may provide a meaningful protective effect. Future prospective studies with more detailed intervention tracking will be needed to confirm this. We also observed that patients with histories of receiving behavioral therapies (such as ABA, physical, occupational, or speech therapy) had higher numbers of psychotropic medications trials on board prior to intake​. This likely reflects the greater clinical complexity of these children – those with more severe or refractory symptoms often require multimodal treatment, engaging multiple services in parallel. It underscores the importance of an integrated approach to care: indeed, evidence from clinical trials supports the combined use of behavioral therapies alongside psychotropic medication to manage challenging behaviors in NDDs [40–42]. Clinically, this suggests that when confronting significant behavioral comorbidities, a combination of therapeutic modalities is often indicated and commonly implemented.

Several important limitations should be considered when interpreting our findings. First, the retrospective, cross-sectional design and reliance on medical record review introduce potential information and selection biases, and they preclude any conclusions about causality. We attempted to control for key demographic covariates, but unmeasured confounding factors (such as varying severity of symptoms, differences in access to care, or other environmental influences) likely remain. Second, our unique cohort – drawn from a single tertiary care neurogenetics clinic within a psychiatry department – limits generalizability. Families seen in this clinic were often specifically seeking neurological or psychiatric evaluation, which may not reflect the broader population of individuals with NDDs who are managed in primary care or other settings. This referral context could bias the sample toward more complex cases or those already deemed in need of psychiatric input. Furthermore, these findings may not fully generalize to countries outside of the United State. In our setting, pediatricians and primary care providers may initiate psychotropic treatment or refer children with developmental concerns directly to early intervention services without specialist consultation. Such pathways may differ from practices in other countries where subspecialist oversight is required for psychiatric treatment, and thus generalizability to non-U.S. healthcare systems may be limited. Next, we were unable to reliably assess the number of prior prescribing clinicians or reconstruct detailed treatment trajectories, including the sequence, duration, or rationale for medication changes. This limits our ability to evaluate how clinical decision-making patterns, or prescribing history may have contributed to polypharmacy.

Additionally, because formal cognitive testing was not routinely available, we were unable to stratify ID severity or include quantitative IQ/DQ measures, which limits our ability to assess how cognitive functioning may relate to psychiatric outcomes. Although a subset of participants had historical testing results such as IQ scores or ADOS evaluations documented from prior evaluations, these data were inconsistently available and not standardized across the cohort. We therefore excluded them from formal analyses to avoid introducing bias due to incomplete ascertainment. This limitation also highlights a broader issue: the categorical neurodevelopmental diagnoses (e.g., ASD, GDD, ID) recorded in the medical record may lack formal reliability, as they were based on clinical impressions rather than systematic, standardized assessments administered uniformly to all participants. This reflects the real-world, retrospective nature of our study and the variability in diagnostic approaches across providers.

Finally, our sample size (*n* = 316) and the need to correct for multiple comparisons may have reduced our power to detect more subtle associations. Some predictors showed trends toward significance that narrowly missed the threshold after correction**​ –** for example, certain internalizing or externalizing symptoms were associated with psychiatric consultation in unadjusted analyses but did not remain significant after false discovery rate adjustment. It is possible that a larger study could confirm these additional associations that we observed as statistical trends. Notwithstanding these limitations, strengths of the study include the integration of genetic and psychiatric data from detailed manual chart review to capture real-world clinical practices.

## Conclusions

In summary, our findings suggest that in patients with GNDDs, psychiatric needs are more closely tied to behavioral comorbidities than to genetic diagnosis status. This has direct clinical implications: clinicians should vigilantly screen for and address co-occurring behavioral problems in children with NDDs regardless of whether a genetic etiology is known and ensure that psychiatric support (including consideration of medication and specialist referral) is guided by the severity of behavioral symptoms rather than genetic labels. The interplay between early developmental interventions and subsequent psychiatric service utilization that we observed warrants further investigation through prospective longitudinal studies across more diverse NDD populations. Such research would help elucidate the temporal relationships between early identification, intervention, and later psychiatric outcomes, clarifying whether robust early intervention can indeed mitigate or delay the emergence of serious psychiatric needs in this vulnerable population. Ultimately, a deeper understanding of these factors will inform clinicians and policymakers in optimizing care pathways – from early screening and referral to integrated treatment – for individuals with NDDs.

## Supplementary Information

Supplementary Material 1.

Supplementary Material 2.

Supplementary Material 3.

Supplementary Material 4.

Supplementary Material 5.

Supplementary Material 6.

## Data Availability

The data sets generated and/or analyzed during the current study are not publicly available because of patient privacy laws but are available from the corresponding author upon request.

## Abbreviations

ABA: Applied behavior analysis
ASD: Autism spectrum disorder
CARING: Care and Research in Neurogenetics
CI: Confidence interval
DD: Developmental delay
EI: Early intervention
FDR: False discovery rate
GDD: Global developmental delay
GNDD: Genetic neurodevelopmental disorder
HIPAA: Health Insurance Portability and Accountability Act
ID: Intellectual disability
IRB: Institutional review board
IRR: Incident rate ratio
NDD: Neurodevelopmental disorder
OR: Odds ratio
OT: Occupational therapy
P/LP: Pathogenic or likely pathogenic
PT: Physical therapy
SES: Socioeconomic status
SIB: Self-injurious behavior
ST: Speech therapy
STROBE: Strengthening the Reporting of Observational Studies in Epidemiology
UCLA: University of California, Los Angeles
