# Supplementary material for: Clinical determinants of psychiatric care in genetic neurodevelopmental disorders: a cross-sectional analysis
Source: J Neurodev Disord. 2025 Oct 7;17:61. doi: 10.1186/s11689-025-09654-0 (PMC12506073; doi:10.1186/s11689-025-09654-0)
Supplement: Supplementary file 1 — Supplementary Material 1. [file 11689_2025_9654_MOESM1_ESM.docx]

Table S1: Descriptive Statistics of Covariates and Factors by Whether Seen by CARING Psychiatry.

|  | **All Patients (*N* = 316)** | | **Seen by CARING Psychiatry (*N* = 127)** | | **Not Seen by CARING Psychiatry (*N* = 189)** | |
| --- | --- | --- | --- | --- | --- | --- |
| **Continuous Covariates** | M | SD | M | SD | M | SD |
| Age at Intake (Months) | 118.99 | 92.69 | 165.96 | 99.77 | 87.43 | 72.34 |
| Area Deprivation Index (National Percentile)^a^ | 9.63 | 11.9 | 10.95 | 13.77 | 8.73 | 10.39 |
|  | **All Patients (*N* = 316)** | | **Seen by CARING Psychiatry (*N* = 127)** | | **Not Seen by CARING Psychiatry (*N* = 189)** | |
| **Categorical Covariates** | N | % | N | % | N | % |
| Sex (Male) | 208 | 65.82 | 89 | 70.08 | 119 | 62.96 |
| Sex (Female) | 108 | 34.18 | 38 | 29.92 | 70 | 37.04 |
| Ethnicity (White) | 178 | 56.33 | 67 | 52.76 | 111 | 58.73 |
| Primary Insurance Status (Commercial) | 217 | 68.67 | 80 | 62.99 | 137 | 72.49 |
| Primary Insurance Status (Public) | 73 | 23.10 | 38 | 29.92 | 35 | 18.52 |
| Primary Insurance Status (No Insurance Documented) | 26 | 8.23 | 9 | 7.09 | 17 | 8.99 |
|  | **All Patients (*N* = 316)** | | **Seen by CARING Psychiatry (*N* = 127)** | | **Not Seen by CARING Psychiatry (*N* = 189)** | |
| **Psychiatric History Factors** | N | % | N | % | N | % |
| Suicidality | 11 | 3.48 | 10 | 7.87 | 1 | 0.53 |
| SIB, Agitated, or Aggressive Behavior | 136 | 43.04 | 77 | 60.63 | 59 | 31.22 |
| Tic or Movement Disorder | 13 | 4.11 | 10 | 7.87 | 3 | 1.59 |
| Internalizing Disorder^b^ | 73 | 23.10 | 46 | 36.22 | 27 | 14.29 |
| Externalizing Disorder^c^ | 71 | 22.47 | 42 | 33.07 | 29 | 15.34 |
| Schizophrenia Spectrum or Other Psychotic Disorder | 7 | 2.22 | 6 | 4.72 | 1 | 0.53 |
| Sleep Issues | 147 | 46.52 | 66 | 51.97 | 81 | 42.86 |
|  | **All Patients (*N* = 316)** | | **Seen by CARING Psychiatry (N = 127)** | | **Not Seen by CARING Psychiatry (N = 189)** | |
| **Neurodevelopmental History Factors** | N | % | N | % | N | % |
| ASD | 207 | 65.51 | 103 | 81.10 | 104 | 55.03 |
| ID | 92 | 29.11 | 48 | 37.80 | 44 | 23.28 |
| DD^d^ | 270 | 85.44 | 99 | 77.95 | 171 | 90.48 |
|  | **All Patients (*N* = 316)** | | **Seen by CARING Psychiatry (N = 127)** | | **Not Seen by CARING Psychiatry (N = 189)** | |
| **Genetic Testing Results Factors^e^** | N | % | N | % | N | % |
| Positive | 152 | 48.10 | 54 | 42.52 | 98 | 51.85 |
| Inconclusive | 41 | 12.97 | 14 | 11.02 | 27 | 14.29 |
| Negative | 53 | 16.77 | 20 | 15.75 | 33 | 17.46 |
| Never Tested | 70 | 22.15 | 39 | 30.71 | 31 | 16.40 |
|  | **All Patients (*N* = 316)** | | **Seen by CARING Psychiatry (N = 127)** | | **Not Seen by CARING Psychiatry (N = 189)** | |
| **Service History Factors** | N | % | N | % | N | % |
| ABA | 132 | 41.77 | 56 | 44.09 | 76 | 40.21 |
| EI | 179 | 56.65 | 54 | 42.52 | 125 | 66.14 |
| PT, OT, or ST | 254 | 80.38 | 94 | 74.02 | 160 | 84.66 |
|  | **All Patients (*N* = 316)** | | **Seen by CARING Psychiatry (N = 127)** | | **Not Seen by CARING Psychiatry (N = 189)** | |
| **Medical Comorbidities Factors** | N | % | N | % | N | % |
| Seizures^f^ | 99 | 31.33 | 36 | 28.35 | 63 | 33.33 |
| Other Medical Comorbidity | 240 | 75.95 | 106 | 83.46 | 134 | 70.90 |
|  | **All Patients (*N* = 316)** | | **Seen by CARING Psychiatry (N = 127)** | | **Not Seen by CARING Psychiatry (N = 189)** | |
| **Family History Factors** | N | % | N | % | N | % |
| ASD, ID, or DD in First-Degree Relative | 51 | 16.14 | 21 | 16.54 | 30 | 15.87 |
| Psychiatric Disorder in First-Degree Relative | 96 | 30.38 | 48 | 37.80 | 48 | 25.40 |

Unless otherwise noted all historical elements were considered binary and were diagnosed prior to clinic intake.

^a^Based on limitations of the Area Deprivation Index, statistics are based on slightly smaller sample sizes for each group.

^b^We defined internalizing disorders to include anxiety disorders, affective disorders, obsessive-compulsive (OCD) and related disorders, gender dysphoria, and eating disorders.

^c^We defined externalizing disorders to include attention-deficit/hyperactivity disorder (ADHD), disruptive, impulse control, or conduct disorders, and substance use disorders (SUDs). Notably, ADHD represented most externalizing disorders in our cohort, with relatively few having SUDs or disruptive, impulse control, or conduct disorders.

^d^For most patients, developmental delays were identified prior to intake. Rarely, patients were seen in very early infancy before developmental delays are easily assessed. In these few patients, this variable was counted as being true for delays that occurred after CARING Clinic intake. This was because clinic intake was defined as the first appointment with a CARING Clinic provider of any specialty and consideration of psychiatric management occurred later in life, when developmental delays had already been identified.

^e^Genetic testing was considered “positive” if at least one P/LP variant was identified. Genetic testing was considered “inconclusive” if only variants that were likely benign or of uncertain clinical significance were identified. Genetic testing was considered “negative” if no variants were reported. We assumed genetic data to be stable over the lifespan and did not make a distinction as to whether genetic diagnosis occurred before or after intake to CARING Clinic.

^f^Serves as a covariate for models that consider medication exposure as the outcome and as an independent variable for models that consider seeing a CARING psychiatrist as the outcome.
