# Supplementary material for: Clinical determinants of psychiatric care in genetic neurodevelopmental disorders: a cross-sectional analysis
Source: J Neurodev Disord. 2025 Oct 7;17:61. doi: 10.1186/s11689-025-09654-0 (PMC12506073; doi:10.1186/s11689-025-09654-0)
Supplement: Supplementary file 2 — Supplementary Material 2. [file 11689_2025_9654_MOESM2_ESM.docx]

Table S2: Chi-Square Analyses Comparing GNDDs and Idiopathic NDDs.

| **Categorical Variable** | **Chi-Square** | **Raw p-value** | **Corrected p-value** |
| --- | --- | --- | --- |
| Seen by CARING Psychiatry | 2.65 | 0.104 | 0.134 |
| Suicidality | 4.09 | 0.043 | 0.086 |
| SIB, Agitated, or Aggressive Behavior | 3.66 | 0.056 | 0.101 |
| Tic or Movement Disorder | 3.4 | 0.065 | 0.106 |
| Internalizing Disorder | 2.67 | 0.102 | 0.134 |
| Externalizing Disorder | 10.69 | 0.001 | 0.003 |
| Schizophrenia Spectrum or Other Psychotic Disorder | 6.95 | 0.139 | 0.167 |
| Sleep Issues | 0.27 | 0.605 | 0.605 |
| ASD | 33.87 | <.001 | 0.001 |
| ID | 2.03 | 0.154 | 0.173 |
| DD † † | 17.88 | <.001 | <.001 |
| ABA | 0.324 | 0.569 | 0.602 |
| EI † | 24.75 | <.001 | <.001 |
| PT, OT, or ST† | 6.26 | 0.012 | 0.027 |
| Seizures† † | 13.94 | <.001 | <.001 |
| Other Medical Comorbidity† | 9.27 | 0.002 | 0.005 |
| ASD, ID, or DD in First-Degree Relative | 3.19 | 0.074 | 0.111 |
| Psychiatric Disorder in First-Degree Relative | 10.06 | 0.002 | 0.005 |
