# Supplementary material for: Clinical determinants of psychiatric care in genetic neurodevelopmental disorders: a cross-sectional analysis
Source: J Neurodev Disord. 2025 Oct 7;17:61. doi: 10.1186/s11689-025-09654-0 (PMC12506073; doi:10.1186/s11689-025-09654-0)
Supplement: Supplementary file 3 — Supplementary Material 3. [file 11689_2025_9654_MOESM3_ESM.docx]

Table S3: Poisson Regression Model Results for Medication Exposure as Outcome.

|  | **Estimate (SE)** | **IRR^a^** | **p-value** | **FDR**  **p-value** |
| --- | --- | --- | --- | --- |
| **Suicidality** |  |  |  |  |
| Yes vs. no | 0.02 (0.19) | 1.02 | 0.9057 | 0.9084 |
| **SIB or Agitation** |  |  |  |  |
| Yes vs. no | 0.48 (0.09) | 1.61 | **<.0001** | **0.0006** |
| **Sleep Disorders** |  |  |  |  |
| Yes vs. no | 0.20 (0.08) | 1.22 | **0.0193** | **0.0452** |
| **Movement Disorders** |  |  |  |  |
| Yes vs. no | 0.17 (0.21) | 1.18 | 0.4275 | 0.5529 |
| **Internalizing Disorders** |  |  |  |  |
| Yes vs. no | 0.25 (0.09) | 1.28 | **0.0087** | **0.0272** |
| **Externalizing Disorders** |  |  |  |  |
| Yes vs. no | 0.39 (0.09) | 1.47 | **<.0001** | **0.0006** |
| **Schizophrenia Spectrum/Psychotic Disorders** |  |  |  |  |
| Yes vs. no | -0.09 (0.20) | 0.91 | 0.6439 | 0.7317 |
| **Autism Spectrum Disorder** |  |  |  |  |
| Yes vs. no | 0.50 (0.10) | 1.65 | **<.0001** | **0.0006** |
| **Intellectual Disability** |  |  |  |  |
| Yes vs. no | 0.25 (0.10) | 1.29 | **0.0111** | **0.0308** |
| **Developmental Delay** |  |  |  |  |
| Yes vs. no | -0.16 (0.12) | 0.85 | 0.1606 | 0.2677 |
| **Early Intervention** |  |  |  |  |
| Yes vs. no | -0.01 (0.09) | 0.99 | 0.9084 | 0.9084 |
| **ABA Therapy** |  |  |  |  |
| Yes vs. no | 0.20 (0.09) | 1.22 | **0.0199** | **0.0452** |
| **PT/OT/ST Services** |  |  |  |  |
| Yes vs. no | 0.37 (0.11) | 1.45 | **0.0011** | **0.0046** |
| **Genetic Results** |  |  |  |  |
| Inconclusive vs. undocumented | 0.16 (0.16) | 1.17 | 0.3127 | 0.4438 |
| Negative vs. undocumented | 0.27 (0.13) | 1.31 | **0.0361** | 0.0694 |
| Positive vs. undocumented | -0.03 (0.12) | 0.97 | 0.7708 | 0.8378 |
| **Relative with Neurodevelopmental Disorder** |  |  |  |  |
| Yes vs. no | -0.33 (0.14) | 0.72 | **0.0227** | **0.0473** |
| **Relative with Psychiatric Disorder** |  |  |  |  |
| Yes vs. no | 0.17 (0.09) | 1.18 | 0.0685 | 0.1223 |
| **Medical/Surgical Comorbidities** |  |  |  |  |
| Yes vs. no | 0.11 (0.11) | 1.12 | 0.3195 | 0.4438 |
| **Psychiatry** |  |  |  |  |
| Yes vs. no | 0.30 (0.09) | 1.36 | **0.0011** | **0.0046** |

All models control for age, sex, race/ethnicity (white vs. other), Area Deprivation Index (national percentile), seizures (yes vs. no), and insurance status (none vs. commercial vs. public).

^a^IRR = incident rate ratio
