# Supplementary material for: Clinical determinants of psychiatric care in genetic neurodevelopmental disorders: a cross-sectional analysis
Source: J Neurodev Disord. 2025 Oct 7;17:61. doi: 10.1186/s11689-025-09654-0 (PMC12506073; doi:10.1186/s11689-025-09654-0)
Supplement: Supplementary file 4 — Supplementary Material 4. [file 11689_2025_9654_MOESM4_ESM.docx]

Table S4: Logistic Regression Model Results for Seeing CARING Psychiatry as Outcome.

|  | **Estimate (SE)** | **OR^a^** | **p-value** | **FDR**  **p-value** |
| --- | --- | --- | --- | --- |
| **Suicidality** |  |  |  |  |
| Yes vs. no | 2.13 (1.08) | 8.44 | **0.0483** | 0.1063 |
| **SIB or Agitation** |  |  |  |  |
| Yes vs. no | 0.86 (0.27) | 2.37 | **0.0013** | **0.0163** |
| **Sleep Disorders** |  |  |  |  |
| Yes vs. no | 0.31 (0.26) | 1.36 | 0.2315 | 0.3858 |
| **Movement Disorders** |  |  |  |  |
| Yes vs. no | 1.87 (0.71) | 6.50 | **0.0081** | 0.0534 |
| **Internalizing Disorders** |  |  |  |  |
| Yes vs. no | 0.73 (0.32) | 2.07 | **0.0238** | 0.0661 |
| **Externalizing Disorders** |  |  |  |  |
| Yes vs. no | 0.75 (0.30) | 2.12 | **0.0126** | 0.0534 |
| **Schizophrenia Spectrum/Psychotic Disorders** |  |  |  |  |
| Yes vs. no | 0.76 (1.19) | 2.14 | 0.5236 | 0.5454 |
| **Autism Spectrum Disorder** |  |  |  |  |
| Yes vs. no | 1.04 (0.30) | 2.83 | **0.0006** | **0.0150** |
| **Intellectual Disability** |  |  |  |  |
| Yes vs. no | -0.25 (0.32) | 0.78 | 0.4244 | 0.4822 |
| **Developmental Delay** |  |  |  |  |
| Yes vs. no | -0.84 (0.35) | 0.43 | **0.0171** | 0.0534 |
| **Early Intervention** |  |  |  |  |
| Yes vs. no | -0.66 (0.27) | 0.52 | **0.0138** | 0.0534 |
| **ABA Therapy** |  |  |  |  |
| Yes vs. no | 0.28 (0.26) | 1.32 | 0.2850 | 0.3958 |
| **PT/OT/ST Services** |  |  |  |  |
| Yes vs. no | -0.28 (0.32) | 0.76 | 0.3935 | 0.4684 |
| **Genetic Results** |  |  |  |  |
| Inconclusive vs. undocumented | -0.49 (0.44) | 0.61 | 0.2638 | 0.3958 |
| Negative vs. undocumented | -0.79 (0.41) | 0.46 | 0.0546 | 0.1063 |
| Positive vs. undocumented | -0.63 (0.33) | 0.53 | 0.0569 | 0.1063 |
| **Relative with Neurodevelopmental Disorder** |  |  |  |  |
| Yes vs. no | 0.32 (0.34) | 1.38 | 0.3422 | 0.4503 |
| **Relative with Psychiatric Disorder** |  |  |  |  |
| Yes vs. no | 0.68 (0.27) | 1.97 | **0.0135** | 0.0534 |
| **Seizures** |  |  |  |  |
| Yes vs. no | -0.75 (0.31) | 0.47 | **0.0152** | 0.0534 |
| **Medical/Surgical Comorbidities** |  |  |  |  |
| Yes vs. no | 0.59 (0.31) | 1.81 | 0.0595 | 0.1063 |
| **Number of CNS Medication Classes** |  |  |  |  |
| One-unit change (increase) | 0.06 (0.07) | 1.07 | 0.3934 | 0.4684 |

All models control for age, sex, race/ethnicity (white vs. other), Area Deprivation Index (national percentile), and insurance status (none vs. commercial vs. public).

^a^OR = odds ratio
