# Supplementary material for: Clinical determinants of psychiatric care in genetic neurodevelopmental disorders: a cross-sectional analysis
Source: J Neurodev Disord. 2025 Oct 7;17:61. doi: 10.1186/s11689-025-09654-0 (PMC12506073; doi:10.1186/s11689-025-09654-0)
Supplement: Supplementary file 5 — Supplementary Material 5. [file 11689_2025_9654_MOESM5_ESM.docx]

Table S5: Number of Patients Exposed to Each Psychotropic Medication Class.

| **Psychotropic Medication Class** | **Exposed** | **%** |
| --- | --- | --- |
| AED/Mood Stabilizer^a^ | 102 | 32.28 |
| Alpha Agonist^a^ | 65 | 20.57 |
| Anticholinergic/Dopamine Agonist | 6 | 1.90 |
| Atypical Antidepressant^a^ | 16 | 5.06 |
| Atypical Antipsychotic^a^ | 77 | 24.37 |
| Barbiturate | 11 | 3.48 |
| Benzodiazepine^a^ | 61 | 19.30 |
| Beta Blocker | 5 | 1.58 |
| First Generation Antihistamine | 13 | 4.11 |
| First Generation Antipsychotic^a^ | 15 | 4.75 |
| Lithium | 5 | 1.58 |
| Melatonin | 47 | 14.87 |
| Muscle Relaxer/Nerve Pain | 3 | 0.95 |
| NET Inhibitor | 12 | 3.80 |
| NMDA Antagonist | 4 | 1.27 |
| Non-Benzodiazepine Anxiolytic | 2 | 0.63 |
| Nootropic | 1 | 0.32 |
| Opioid^a^ | 1 | 0.32 |
| Opioid Antagonist | 2 | 0.63 |
| Sedative/Hypnotic^a^ | 2 | 0.63 |
| SNRI | 4 | 1.27 |
| SSRI^a^ | 71 | 22.47 |
| Stimulant^a^ | 57 | 18.04 |
| TCA | 3 | 0.95 |
| Triptan | 1 | 0.32 |

Summary of patients exposed to each of the 25 psychoactive medication classes.

^a^Represents classes with patients who were exposed to multiple medications within the same class.
