# Supplementary material for: Clinical determinants of psychiatric care in genetic neurodevelopmental disorders: a cross-sectional analysis
Source: J Neurodev Disord. 2025 Oct 7;17:61. doi: 10.1186/s11689-025-09654-0 (PMC12506073; doi:10.1186/s11689-025-09654-0)
Supplement: Supplementary file 6 — Supplementary Material 6. [file 11689_2025_9654_MOESM6_ESM.docx]

Table S6: Exposures by Medication.

| **Medication** | **Exposed** | **%** |
| --- | --- | --- |
| ***AED/Mood Stabilizer^a^*** | | |
| Adrenocorticotrophic Hormone (ACTH) | 2 | 0.63 |
| Cannabidiol | 1 | 0.32 |
| Carbamazepine | 10 | 3.16 |
| Ethosuximide | 2 | 0.63 |
| Felbamate | 5 | 1.58 |
| Gabapentin | 9 | 2.85 |
| Lacosamide | 5 | 1.58 |
| Lamotrigine | 36 | 11.39 |
| Levetiracetam | 40 | 12.66 |
| Oxcarbazepine | 19 | 6.01 |
| Perampanel | 1 | 0.32 |
| Phenytoin | 2 | 0.63 |
| Rufinamide | 4 | 1.27 |
| Topiramate | 14 | 4.43 |
| Valproate Product | 46 | 14.56 |
| Vigabatrin | 5 | 1.58 |
| Zonisamide | 10 | 3.16 |
| ***Alpha Agonist^a^*** | | |
| Brimonidine | 1 | 0.32 |
| Clonidine | 26 | 8.23 |
| Guanfacine | 47 | 14.87 |
| ***Anticholinergic/Dopamine Agonist*** | | |
| Amantadine | 1 | 0.32 |
| Benztropine | 5 | 1.58 |
| ***Atypical Antidepressant^a^*** | | |
| Bupropion | 4 | 1.27 |
| Mirtazapine | 4 | 1.27 |
| Trazodone | 9 | 2.85 |
| ***Atypical Antipsychotic^a^*** | | |
| Aripiprazole | 35 | 11.08 |
| Asenapine | 2 | 0.63 |
| Clozapine | 2 | 0.63 |
| Lurasidone | 1 | 0.32 |
| Olanzapine | 13 | 4.11 |
| Paliperidone | 5 | 1.58 |
| Quetiapine | 18 | 5.7 |
| Risperidone | 54 | 17.09 |
| Ziprasidone | 6 | 1.9 |
| ***Barbiturate*** | | |
| Phenobarbital | 11 | 3.48 |
| ***Benzodiazepine^a^*** | | |
| Alprazolam | 2 | 0.63 |
| Clobazam | 12 | 3.8 |
| Clonazepam | 23 | 7.28 |
| Clorazepate | 2 | 0.63 |
| Diazepam | 22 | 6.96 |
| Lorazepam | 15 | 4.75 |
| Midazolam | 1 | 0.32 |
| Temazepam | 3 | 0.95 |
| Unspecified Benzodiazepines | 1 | 0.32 |
| ***Beta Blocker*** | | |
| Metoprolol | 3 | 0.95 |
| Propranolol | 2 | 0.63 |
| ***First Generation Antihistamine*** | | |
| Cyproheptadine | 2 | 0.63 |
| Diphenhydramine | 9 | 2.85 |
| Hydroxyzine | 2 | 0.63 |
| ***First Generation Antipsychotic^a^*** | | |
| Chlorpromazine | 11 | 3.48 |
| Haloperidol | 4 | 1.27 |
| Pimozide | 1 | 0.32 |
| Thioridazine | 1 | 0.32 |
| ***Lithium*** | | |
| Lithium | 5 | 1.58 |
| ***Melatonin*** | | |
| Melatonin | 47 | 14.87 |
| ***Muscle Relaxer/Nerve Pain*** | | |
| Baclofen | 1 | 0.32 |
| Pregabalin | 1 | 0.32 |
| Tizanidine | 1 | 0.32 |
| ***NET Inhibitor*** | | |
| Atomoxetine | 12 | 3.8 |
| ***NMDA Antagonist*** | | |
| Memantine | 4 | 1.27 |
| ***Non-Benzodiazepine Anxiolytic*** | | |
| Buspirone | 2 | 0.63 |
| ***Nootropic*** | | |
| Piracetam | 1 | 0.32 |
| ***Opioid^a^*** | | |
| Hydrocodone/Acetaminophen | 1 | 0.32 |
| Oxycodone | 1 | 0.32 |
| ***Opioid Antagonist*** | | |
| Naltrexone | 2 | 0.63 |
| ***Sedative/Hypnotic^a^*** | | |
| Eszopiclone | 1 | 0.32 |
| Zolpidem | 2 | 0.63 |
| ***SNRI*** | | |
| Venlafaxine | 4 | 1.27 |
| ***SSRI^a^*** | | |
| Citalopram | 12 | 3.8 |
| Escitalopram | 16 | 5.06 |
| Fluoxetine | 24 | 7.59 |
| Fluvoxamine | 4 | 1.27 |
| Paroxetine | 8 | 2.53 |
| Sertraline | 27 | 8.54 |
| ***Stimulant^a^*** | | |
| Dextroamphetamine/Amphetamine Product | 32 | 10.13 |
| Lisdexamfetamine | 10 | 3.16 |
| Methylphenidate Product | 66 | 20.89 |
| Pemoline | 1 | 0.32 |
| Unspecified Stimulant | 1 | 0.32 |
| ***TCA*** | | |
| Amitriptyline | 1 | 0.32 |
| Clomipramine | 2 | 0.63 |
| ***Triptan*** | | |
| Rizatriptan | 1 | 0.32 |

Summary of patient exposures to each medication within the CNS active classes. “Exposed” refers to any time prior to date of intake.

^a^Refers to classes where patients had tried or were taking multiple medications within the same class (i.e., intraclass polypharmacy).
